# Application of Genomic Data for Reliability Improvement of Pig Breeding Value Estimates

**DOI:** 10.3390/ani11061557

**Published:** 2021-05-27

**Authors:** Ekaterina Melnikova, Artem Kabanov, Sergey Nikitin, Maria Somova, Sergey Kharitonov, Petr Otradnov, Olga Kostyunina, Tatiana Karpushkina, Elena Martynova, Aleksander Sermyagin, Natalia Zinovieva

**Affiliations:** 1 L.K. Ernst Federal Research Center for Animal Husbandry, Dubrovitzy Estate, Podolsk District, Moscow Region, 142132 Podolsk, Russia; akabanov@vij.ru (A.K.); snikitin@vij.ru (S.N.); somova-mm@yandex.ru (M.S.); kharitonovsn@vij.ru (S.K.); deriteronard@gmail.com (P.O.); kostolan@yandex.ru (O.K.); tati.kriz@gmail.com (T.K.); alex_sermyagin85@mail.ru (A.S.); n_zinovieva@mail.ru (N.Z.); 2 Center of Life Sciences, Skolkovo Institute of Science and Technology, 3, ul. Nobelya, 143026 Moscow, Russia; elenamartynovaster@gmail.com

**Keywords:** pigs, estimated breeding value, genomic prediction, genomic evaluation, ssGBLUP, reliability of genomic prediction

## Abstract

**Simple Summary:**

Selection of pigs in Russia is carried out within the framework of separate large holdings. Such a system does not allow for the use of sufficiently large amounts of data (on all individuals of the breed) to obtain the most reliable breeding value estimates. This problem is especially relevant for low-inherited reproduction traits (for example, prolificacy), which are the main ones for maternal pig breeds. In this regard, our study considered the possibility of improving the accuracy of the breeding value assessment of Large White pigs (replacement pigs, sows and boars) through the use of genomic data obtained on a high-density hybridization chip.

**Abstract:**

Replacement pigs’ genomic prediction for reproduction (total number and born alive piglets in the first parity), meat, fatness and growth traits (muscle depth, days to 100 kg and backfat thickness over 6–7 rib) was tested using single-step genomic best linear unbiased prediction ssGBLUP methodology. These traits were selected as the most economically significant and different in terms of heritability. The heritability for meat, fatness and growth traits varied from 0.17 to 0.39 and for reproduction traits from 0.12 to 0.14. We confirm from our data that ssGBLUP is the most appropriate method of genomic evaluation. The validation of genomic predictions was performed by calculating the correlation between preliminary GEBV (based on pedigree and genomic data only) with high reliable conventional estimates (EBV) (based on pedigree, own phenotype and offspring records) of validating animals. Validation datasets include 151 and 110 individuals for reproduction, meat and fattening traits, respectively. The level of correlation (r) between EBV and GEBV scores varied from +0.44 to +0.55 for meat and fatness traits, and from +0.75 to +0.77 for reproduction traits. Average breeding value (EBV) of group selected on genomic evaluation basis exceeded the group selected on parental average estimates by 22, 24 and 66% for muscle depth, days to 100 kg and backfat thickness over 6–7 rib, respectively. Prediction based on SNP markers data and parental estimates showed a significant increase in the reliability of low heritable reproduction traits (about 40%), which is equivalent to including information about 10 additional descendants for sows and 20 additional descendants for boars in the evaluation dataset.

## 1. Introduction

The inclusion of genomic data in animal evaluation procedure provides undeniable advantages in comparison with traditional methods, which use information about animal relationships and productivity results only [1,2]. The first of them is the earlier selection of candidates due to the differentiation of siblings’ breeding value estimates before obtaining their own phenotypic data, which is one of the most pressing issues in pig breeding [3]. So, the selection of boars in most cases is carried out after receiving their own productivity results of meat, fattening and growth traits. This assessment is calculated after the selection candidates reach a live weight of 100 kg (on average after 150–170 days of life). In this case, the use of genomic prediction (genomic breeding value, GEBV) as a selection criterion would significantly reduce the cost of raising additional replacement individuals.

The second element is to increase the accuracy of animals’ breeding value estimates of traits with low heritability, such as reproduction traits (sow productivity). In pig breeding, selection programs for improving animals of maternal breeds are mainly focused on genetic changes in the population based on the characteristics of reproduction (prolificacy). Estimates of animals (both boars and sows) for these traits, even with offspring data, have a relatively low level of reliability. This is a limiting factor that reduces the effectiveness of breeding program. Thus, the additional use of genomic data is intended to increase the accuracy of breeding value estimates by these traits, thereby making the selection criteria more objective [3,4,5].

The accuracy of GEBV depends on some main parameters, in particular the level of linkage disequilibrium (LD) between markers and quantitative trait loci (QTL); the accumulated data size of production records; the quality of pedigree and phenotype measurements; the number of genotyped animals; trait heritability; the actual distribution of QTL effects; and the optimality of the statistical model [4,6,7,8,9]. Implementation of genomic selection to livestock breeding practice gives rise to a large number of difficult issues. The list of such issues includes: addition of genomic information in the national genetic assessment system, expanding the reference subpopulation, the management of long-term genetic progress and inbreeding based on genomic data, and problems related to performing calculations [8,10,11].

Currently, a number of approaches have been developed to calculate genomic breeding value estimates: GBLUP (with the inverse of the convenient relationship matrix replaced by the inverse of the genomic relationship matrix G^−1^) [7,8], Bayesian methods (using different genetic variances for each SNP, or assuming that some SNPs have effects following a t-distribution and others have zero effects, or assuming that some SNPs have zero effects and others to follow a normal distribution and etc.) [1,9,12,13] and ssGBLUP (using the H matrix which combines the A matrix derived from pedigree and G matrix from genomic data) [14]. Each of these methods has its own advantages and assumptions. However, one of the most proven, convenient and reliable methods is the ssGBLUP method, which provides the use of the entire accumulated volume of animal data in a single procedure [15]. The main advantage of ssGBLUP in comparison with GBLUP is that it allows EBV assessment of both genotyped and non-genotyped animals in a single procedure through hybrid matrix usage (combining probabilistic and genomic relationships) as a covariance structure in a mixed model [14,16,17]. Song H. et al. [18] obtained genomic estimates of Yorkshire pigs using GBLUP, Bayesian mixed model (BayesR) and ssGBLUP. For reproduction and growth traits, ssGBLUP showed higher accuracy than GBLUP and BayesR. In addition, the ssGBLUP method has a lower bias of the estimates.

At the same time, the ssGBLUP is not inferior to multi-stage methods in accuracy of estimates obtained. The method allows for more complex models to be applied and also automatically defines weights of all sources of information for the final breeding value [16,19]. The advantages of the ssGBLUP method are also noted by many authors regarding the genomic evaluation in different species [17,19,20,21,22].

Selection in pig breeding in the Russian Federation is built within the framework of separate large agricultural holdings with a closed cycle of genetic resources reproduction. In this regard, the organization of purebred populations’ pig breeding is based on the main traits of phenotypes data collection, the individual breeding value assessment, selection of individuals in breeding groups and future generation parents’ selection. The overall effectiveness of the entire system depends on the accuracy of each of these elements. The inclusion of genomic evaluation in the system implies additional costs incurred by the business. In this regard, there are a number of questions about the economic feasibility of implementing such a method [23,24]. Since genomic prediction is an addition to the conventional assessment of breeding value, it is necessary to plan in advance the payback period of obtaining animals’ genomic data (sample collection, genotyping and data processing) and reference population size needed for sufficient assessment accuracy and effectiveness of genomic prediction for traits due to reference population size. It is necessary to optimize the pig breeding program, taking into account the annual cost of genotyping a part of the herd and estimating profit from reducing the cost of raising by decreasing of selection candidates’ number [25]. This is especially important because of breeding stock maintaining and growing cost increase [26]. When using genomic selection of young animals (pedigree- and genomic data- based GEBV), the tested traits should have high economic value. According to Tribout et al. (2013) and Lillehammer et al. (2011), the potential improvements provided by genomic data application, especially for reproduction traits and maternal effects, significantly outweigh the benefits of increasing phenotypic datasets for these indicators [3,5].

An important practical stage in genomic selection implementation is the validation of genomic predictions based on correlation and regression analysis with highly reliable breeding value estimates [27] or adjusted phenotypes [18]. A high level of estimates correlation indicates the consistency of the genomic prediction, and the proximity of the regression coefficient to unity shows the absence of estimates bias (inflation).

Possibilities of the genomic data application for the needs of livestock breeding are also associated with a number of areas. These are the development of low-density SNP chips (less than 3000 markers) [28], which can significantly reduce the genotyping cost per animal; the preliminary analysis and selection of QTLs causing the majority of trait’s genetic variation [13,29,30]; and the development of methods allowing the use of a large number of genotyped animals’ genomic data [19,31].

The objectives of our study are to test the ssGBLUP approach for genomic prediction of replacement pigs’ main selection traits breeding values in two different productivity aspects. The first is to assess the genomic data contribution to reliability improvement of low-heritable reproduction traits’ EBV (total number and number of born alive piglets on litter in the first parity). The second is to determine the effectiveness of replacement animals’ genomic selection by meat and fattening traits (muscle depth, days to 100 kg and backfat thickness over 6–7 rib).

## 2. Materials and Methods

### 2.1. Phenotypes

Data about Large White pigs’ phenotypic measurements of meat, growth and repro-duction traits were used. The information about individuals born between 2015 and 2020 was included in the data sets. The assessment was performed in terms of meat and fatness productivity according to the following characteristics: backfat thickness over 6–7 ribs (BF1, mm), muscle depth (MD, mm) and days to 100 kg (Age, days). The following reproductive qualities were taken into account: the number of all piglets born (TNB), and the number of piglets born alive (NBA) based on the results of the first farrowing.

The data set on meat and growth traits had 41,941 records, of which 5445 were boars and 36,496 were sows. The reproduction data set comprised 9433 records of first sow farrowing (Table 1).

### 2.2. Genotypes

A high-density GGP Porcine HD hybridization chip (Illumina/Neogen, Lincoln, NE, USA) with 70,000 SNPs for all major pig breeds (an average interval between markers of ~42 kb, 20 key causative mutations) was used for boars and sows genotyping. Genome-wide genotyping data was presented in the form of 1483 individual genotypes from the GenomeStudio 2 software (Illumina Inc., San Diego, CA, USA).

The following were excluded from genomic data: SNPs with an accuracy (GC Score, GT Score) less than 0.2; animal records with a genotype absence rate of more than 10%; SNP markers absent in more than 10% of genotyped animals; SNP markers with a minor allele frequency of less than 5%; SNP markers deviating from the Hardy-Weinberg equilibrium, with the threshold *p*-value set to 10^−6^; and animal records with Mendelian errors in allele inheritance. The presence of such errors is treated as errors in the records of animals’ pedigree.

### 2.3. Statistical Analyses and Evaluation

Statistical processing of phenotypic data was carried out using the STATISTICA 10 package. Pedigree records were analyzed using the CFC software package [32]. Primary processing and conversion of genomic data were carried out using an integrated development environment (IDE) RStudio for the R programming language. Genomic data from the final report format were converted into “. ped” and “. map” files for further processing in the PLINK software package [33]. The estimates of individuals’ breeding value (EBV, GEBV) were calculated using BLUP AM and ssGBLUP methodology with BLUPF90 software package [34]; the genetic variance and traits’ covariance were estimated using the restricted maximum likelihood method in the REMLF90 software [35]. In matrix notation, the mixed linear model for genetic predictions on the BLUP AM is:

y = Xb + Za + e,

where y is the vector of phenotype observations on trait (TNB, NBA, BF1, MD and Age100); b—vector of fixed effects («farm-year—contemporary group» by date of measurement; «sex of the animal»; «weight of the animal»—for meat, fatness and growth traits; «farm-year—contemporary group» by farrow date—for reproduction traits); a—vector of random animal effect; e—vector of random residual effect; X, Z—incidence matrices relating records to fixed and random effects, respectively.

The fixed effects significance on the variability of the dependent variable was evaluated on exact Fisher criterion basis. Models with other sets of random effects (for example, with an additional service sire effect for reproduction traits) were tested with the Akaike criterion, resulting in optimal models usage in the study.

Calculation of genomic relationship matrix G was carried out in BLUPF90 package using a linear method proposed by VanRaden [6]. To overcome problem with inversion of matrix G for genotyped animals, G matrix was modified as *G = α G + β A*_22_, where the coefficients α = 0.95 and β = 0.05 (by default in BLUPF90 package). To reduce bias in GEBV, we use the default in BLUPF90 procedure where

$$\bar{diagA_{22}}=\bar{diagG},\;\bar{offdiagA_{22}}=\bar{offdiagG}.$$

In the ssGBLUP model, the relationship matrix (*A*^−1^) is replaced by *H*^−1^, which uses both pedigree and genomic information [14]. The fixed and random effects in the ssGBLUP model are exactly the same as those in the BLUP AM model. H matrix is a relationship matrix with *H*^−1^ constructed as [16]:

$$H^{-1}=A^{-1}+\left[\begin{array}{cc} 0 & 0\\ 0 & \tau G^{-1}-\omega A_{22}^{-1}\; \end{array}\right],$$

where *A* is the pedigree-based relationship matrix for both genotyped and non-genotyped animals, *A*_22_^−1^ is the inverse of a pedigree-based relationship matrix for genotyped animals only, *G* is the genomic relationship matrix adjusted to the same scale as *A*_22_ and *τ* = *ω* = 1 (by default) [15].

The reliabilities of EBV(GEBV) were calculated according to standard error, as follows:

Rel = 1 − PEV/varA,

where PEV is the prediction error variance and varA is the additive genetic variance.

## 3. Results

The reference group of individuals, information about which formed the genomic database, was represented by 1194 individuals, born in 2015–2019, with information on 46,277 SNP markers. The structure of the pedigree data of the studied population of pigs and the reference group of genotyped individuals is shown in Table 2.

### 3.1. Datasets for Validating Genomic Estimates

The validation dataset for reproduction traits was constructed from whole dataset (WD) without validating animals’ records. Validating animals were ones with the estimates reliability (Rel_EBV_) that exceeded the average level for the entire studied population (Rel > 0.36). The group of validating animals included 3 boars (mean Rel = 0.59) and 148 sows (mean Rel = 0.41). For those, phenotypic information was excluded from the phenotypic datasets (for sows), and information on the relationship between validating animals and their offspring (189 boar offspring and 548 sow offspring) was removed from the pedigree. On the formed set basis (partial dataset for reproduction traits (PD_repr_)), the validating animals’ breeding value was estimated using the ssGBLUP method with information on 1194 genotyped animals (reference group).

Validation of genomic assessments for meat and fattening qualities was carried out similar to described procedure. For this, partial dataset (PD_meat_) was formed, in which 110 animals (10 boars and 100 sows) were selected, the reliability of the EBV estimates (Rel) exceeded the threshold value of 0.8 (for boars, the average Rel = 0.82, the number offspring 718; for sows the average Rel = 0.83, the number of offspring 2966). The phenotypic records of these individuals were removed from the dataset, and the relationships between the validated animals and their offspring were set to null in the pedigree.

Thus, for validating animals, some estimates were obtained from a whole (WD) and partial (PD) data sets: parental average (PA_PD_), genomic prediction based on pedigree and genotype (GEBV_PD_), genetic estimates on pedigree, own phenotype and offspring record (EBV_WD_) and genomic EBV based on all available information (GEBV_WD_).

In the study, we evaluated the variance components (additive and residual variances by traits) and compared their values, calculated using only pedigree data and combined information about the relationship of individuals (pedigree + genomic data) (Table 3).

The analysis shows a slight shift in the indicators of variances and heritability coefficients by traits (from 2 to 8%). Such results characterize, on the one hand, the relatively low contribution of genomic data to the clarification of the relationship between all individuals (the share of genotyped individuals in the total sample is about 3% for meat and 12% for reproduction traits), taken into account when calculating estimates. On the other hand, this indicates an adequate level of consistency between the values in the probabilistic relationship matrix (matrix A) and the matrix that considers the already realized relationship (matrix H) between individuals. This provides the basis for using genomic data in assessing (re-evaluating) and predicting the breeding values of individuals in the study sample.

The analysis of inbreeding coefficients calculated on pedigree data and pedigree-genomic information (diagonal elements matrix A and matrix H) indicates that the probabilistic values are refined by including information about the genome. All values of the matrix A diagonal elements were equal to 1 (outbred animals), the average value of the diagonal elements = 1; for the matrix G (genomic relationship matrix), these values ranged from 0.83 to 1.31 with an average of 0.97.

### 3.2. Validation of Genomic Estimates of Breeding Value

To test the effectiveness of genomic data in refining the young animals’ prediction of breeding value without phenotype and offspring data, the estimates obtained by the ssGBLUP method were compared with the final EBV_WD_ and GEBV_WD_ values obtained from all available information sources.

One of the genomic estimates’ reliability criterion is their correlation with high reliable estimates of breeding value [27]. The “consistency” values of these indicators for the validated animals are shown in Table 4.

Thus, for the samples of validating animals, relatively high indicators of comparability of estimates were obtained using “whole data” on the one hand and pedigree and genome data on the other hand (partial data), which provides a reason to assume the consistency of the obtained GEBV_PD_ and GEBV_WD_ values.

The final estimates (EBV_WD,_ GEBV_WD_) are a kind of reference, because they have the highest reliability. Correlation between pairs of estimates EBV_WD_ and PA_PD_ had higher values (r = + 0.39…+ 0.82) than correlation between EBV_WD_ and GEBV_PD_ (r = + 0.51…+ 0.77) for all traits. That is, the pedigree-based prediction is more accurate with respect to the final genetic estimates compared to genomic predictions. However, pedigree-based scores are worse at predicting the final genomic values compared to genomic predictions. At the same time, the genomic estimates conformity (GEBV_PD_ and GEBV_WD_) is higher (r = +0.60…+0.90) than the pedigree-genetic estimates conformity (PA_PD_ and EBV_WD_) (r = +0.39…+0.82). Thus, higher correlation coefficients of genomic estimates (preliminary and final) in comparison with genetic ones indicate the superiority of the former. This is also confirmed by the highest level of confidence in the relationship between these estimates, which will be shown below.

Correlation between predictions and final estimates for meat and fattening qualities had a lower level than for reproduction traits. It is important to note that the genomic estimates obtained on partial and whole datasets are consistent at medium (for meat traits) and high levels (for reproduction traits), which allows one to count on an acceptable level of animal selection efficiency based on the GEBV_PD_.

**Figure 1:**
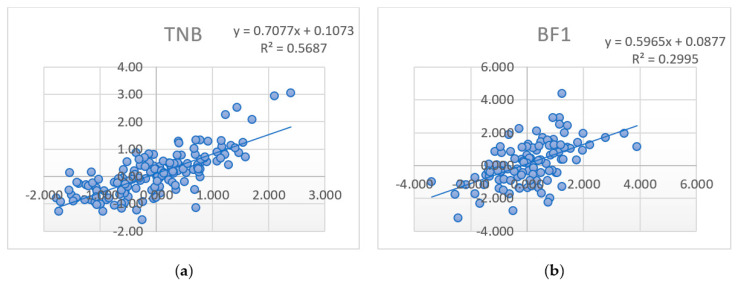
Dynamics of EBV_WD_ depending on GEBV_PD_ for (**a**) TNB and (**b**) BF1 traits of the validated animals.

Of particular interest is the measure of changes in estimates (regression) EBV_WD_ and GEBV_WD_ depending on the predictions’ growth (PA_PD_, GEBV_PD_). In some studies [27], the regression of high reliable EBV to GEBV is considered as “inflation” (loss of significance, bias) (Figure 1a,b).

The inflation was defined by regression with the final high reliable estimates (EBV_WD,_ GEBV_WD_) on the preliminary predictions (PA_PD_, GEBV_PD_) and final genomic (GEBV_WD_) estimates (Table 5). Thus, regression coefficients (b) based on parent averages were the highest for TNB and NBA traits (b_repr_ = 1.03…1.06), confirming the significant contribution of kinship relationships to the final assessment of individuals based on the BLUP AM procedure. At the same time, these indicators for meat qualities varied within the limits of b_meat_ = 0.54…0.93. The determination coefficients (R^2^) that characterize the prediction reliability [2] were at a relatively low level and varied within 0.16…0.67. The lowest values of the coefficients (b and R^2^) were obtained by analyzing the GEBV_PD_ estimates: b_repr_ = 0.59…0.71 (for reproduction traits) and b_meat_ = 0.50…0.65 (for meat and growth traits); R^2^ varied between 0.17 and 0.57.

The determination coefficient (R^2^) characterizes prediction reliability [2]. Relatively low values of R^2^ were obtained by analyzing the regression of both final estimates on PA_PD_: R^2^_repr_ = 0.55…0.67 (for reproduction traits), R^2^_meat_ = 0.15…0.37 (for meat and growth traits) and conventional EBV_WD_ on GEBV_PD_: R^2^_repr_ = 0.48… 0.57 (for reproduction traits), R^2^_meat_ = 0.17…0.30 (for meat and growth traits). Similar to the correlation coefficients, the determination coefficients’ highest values were for GEBV_PD_ and GEBV_WD_ for all traits (R^2^ = 0.36…0.81).

Thus, the results show that preliminary genomic estimates have the greatest predictive power (reliability) in relation to the final genomic breeding values. Inclusion of genomic data increases the animals’ breeding value accuracy and sustainability in comparison to the conventional estimates (BLUP AM). Correlations between final genetic and genomic estimates (EBV_WD_, GEBV_WD_) by traits were: +0.94, +0.91 and +0.93 for BF1, MD and Age100, respectively, and +0.89 for both TNB and NBA. The change in the final estimates due to the inclusion of genomic data was more noticeable for low-inherited reproduction traits. In this regard, the reliability of the final genomic breeding value estimates is particular important.

### 3.3. Improving the Accuracy of Breeding Value Estimates

Improving the accuracy of breeding value estimates is particularly relevant for reproduction indicators because reproduction traits have a relatively low heritability and can be measured exclusively on female individuals. Thus, in the study population, according to the TNB and NBA traits, the reliabilities of both dam and boar estimates were quite low (Table 6). The pedigree-based prediction has a confidence level of 0.26% for sows. The obtained EBV of boars was based solely on their offspring records, that is, on the results of their daughters’ first farrowing. The average reliability of their estimates obtained using the BLUP AM methodology in the presence of 1 to 50 first-time farrowing daughters is 0.26%. Sires are proven (Rel > 0.70) if there are at least 50 descendants. An estimate based on 200 offspring data allows for the estimation of boars with a Rel > 0.80, while adding genomic data to this number of daughter records raises this figure to 0.90.

For sows, the genomic data inclusion in the calculation (even before the first farrowing data is obtained) increases the reliability of the estimation. The reliability of the estimates surpasses of the assessment by pedigree + own phenotype, allowing animals to get values comparable to the assessment results based on three sources of information (pedigree, own phenotype, offspring records (from 1 to 10)).

For meat and fattening indicators, the reliability of estimates for pedigree and genome is at the level of estimates for pedigree and own phenotype.

### 3.4. Selection of Individuals Based on Genomic Prediction

The main advantage of genomic prediction over pedigree estimation is the ability to differentiate the breeding value of complete siblings. Scores based on the parent averages will be the same for all descendants from the same parent pair. At the same time, genomic prediction allows one to select the most promising animals guided by the total value of the SNP effects-provided correction. Thus, when forming a group of replacement young animals, even before weaning piglets in the same litters, piglet leaders can be identified, characterized by the most desirable indicators of GEBV. In this case, the element of genomic assessment of meat and fattening qualities allows one to reduce the cost of raising all piglets from one litter to 100 kg (even before obtaining their phenotype information) via the most promising individuals’ early selection in the herd replacement groups.

To be certain of animals’ selection based on genomic estimates’ (GEBV) implementation superiority in comparison with the selection by parental averages (PA), the average means of the groups’ formed on these criteria were compared. The intensity of selection was 50%, that is, for each of the criteria, the animals were selected according to their excess over the average value for the entire group. The results are presented in Table 7.

The average EBV analysis for groups of individuals indicates a greater efficiency of selection based on genomic estimates for meat and fattening qualities. The superiority of the group formed by GEBV is 66%, 22% and 24% in terms of “fat thickness over 6–7 thoracic vertebrae”, “muscle depth” and “days to 100 kg”, respectively. Simultaneously, the selection of the characteristics of reproduction in terms of efficiency did not have significant differences when using PA and GEBV estimates as a criterion. In our opinion, this is due to the low heritability and repeatability of both breeding value estimates for these traits (PA and GEBV).

## 4. Discussion

Genomic selection significantly changes the entire system of animal breeding. However, the accuracy of genomic selection itself is determined by many factors, such as the size of the reference population, the density of markers, the heritability of the trait, the effective population size, and the distribution of QTL effects [36,37]. Achieving acceptable genomic prediction accuracy for replacement of young individuals requires the creation of a large reference population of genotyped animals with phenotypic records. This is especially relevant for evaluating traits with low heritability, which in our study are represented by reproduction traits.

Thus, since the development of the theoretical basis and the creation of the first reference groups of genotyped animals, an impressive amount of data on the genotyped individuals has been accumulated. Creating a reference population problem was described by H. Song et al., 2017 [18]. They considered the effectiveness of using a mixed reference population based on three samples of the Yorkshire pig breed, and it was proved that the inclusion in the reference population of individuals that are not related or weakly related genetically to the candidates for selection is impractical, since it increases the bias of estimates and reduces their accuracy. In the Ostersen et al. (2016) study on three pig breeds, the selection core group members from total number of genotyped animals were compared (using algorithm for proven and young animals in ssGBLUP procedure) [19]. The authors identified the most optimal criteria for the creation of core group that resulted in more accurate EBV. As a reference group, our study used purebred individuals of a Large White breed (1194 heads) of one breeding organization (5 farms) born between 2015 and 2018. The reference group included parents and full and half-siblings, for which the values of genomic predictions were later determined.

Several studies are devoted to calculating the most accurate (objective) genomic relationship matrix [13,14,16,38,39]. Thus, S. Forni et al. (2011) compared different methods of relationship matrix construction. The authors concluded that distortions in genomic predictions might be associated with an incorrect weighting of polygenic and genomic components. Using pedigree-genome relationship matrix (used in the ssGBLUP procedure) allows one to take this limitation into account [39]. In this regard, in our work, the ssGBLUP procedure was used without additional relationship matrix adjustment (H). The minimum and maximum values of the diagonal elements of the matrix A (pedigree matrix) were 1, and the average value of the diagonal elements = 1; for the matrix G (genomic relationship matrix), these values ranged from 0.83 to 1.31, and the average value was 0.97.

Heritability coefficient estimates by traits, calculated using pedigree and genomic data, in our study were in the range of 2–8%. Similar values of both the levels of heritability and their limits of change due to the inclusion of information about genomes were obtained in the studies of J. Hidalgo et al. (2020) [40]. Changes in heritability of fecundity depending on the type of information about relationships based on genomic data (pedigree) were 0.09–0.06 (0.08–0.09), and growth traits in pigs were 35.10–16.50 (32.50–23.70). This fact allowed the authors to assume that in populations undergoing genomic selection, the variance components estimated without genomic information may be biased [40]. However, the authors noted that the decrease in genetic variance observed over time under the influence of directed selection is reflected in both heritability indicators obtained using pedigrees alone and including genomic information in a similar way (based on trend analysis). The study of S. Forni et al. (2011) also noted a slight change in the litter size additive and residual variance (by 1–3%) when calculation was performed on pedigree data only and with genome information. The authors concluded that the estimates of additive genetic variance with pedigrees or joint pedigree-genomic relationships are similar when the differences between the average diagonal and average non-diagonal elements in the matrix G (developed on genomic data) are similar to those in matrix A (calculated on pedigree data) [39].

In the Veerkamp et al. (2016) study on Holsten-Friesian cattle, a comparison of the genomic relationship matrices’ (GRM) (full sequence data and preselected SNP from genome-wide association) usage effectiveness analysis was made. The authors found that when selected variants were used, accuracy of genomic predictions decreased and the proportion of total variance explained was considerably smaller [29]. At the same time, Fragomeni et al. (2017) on simulated data concluded that using weighted genomic relationship matrix in single-step GBLUP procedure can account for causative quantitative trait nucleotides (QTN) and accuracy of genomic breeding values increased from 0.49 to 0.99 [30].

For the population we studied, the refinement of the variance estimates was quite low for all the analyzed traits, which may be due to the limited genotyped reference group of individuals and a slight change in the indicators (relative to the entire data set) of the relationship based on genomic data. Therefore, the change was 8% for the reproduction traits and 3–6% for the meat and fatness traits.

There are currently two main ways for accuracy of genomic prediction’s determining: cross-validation and verification based on high-precision estimates of breeding value. Cross-validation is performed on multiple evaluation of genomic predictions obtained with “*n*” subsets of data formed from the complete set and correlates these predictions with the observed values (adjusted phenotypes). The second verification option involves obtaining genomic estimates for animals with high accuracy of conventional EBV [37]. In our study, the second variant of genomic prediction’s validation was used. It confirmed an acceptable level of correlation between final EBV_WD_ and GEBV_PD_ scores (r varied from +0.51 to +0.55 for meat and fattening qualities, and from +0.75 to +0.77 for reproduction traits). Regression coefficients (bias index) ranged from 0.50 to 0.65 for meat and fattening qualities and from 0.59 to 0.71 for reproduction traits. Similar values of the accuracy and bias of genomic estimates were obtained in the study of Song et al. (2017). Accuracy for the backfat thickness was +0.49, for days to 100 kg varied from +0.49 to +0.53, and for the reproduction traits from +0.53 to +0.59; unbiasedness for meat and fattening qualities varied from 0.65 to 0.88 and for the reproduction traits from 0.78 to 1.00.

It is worth noting that the correlation between final genetic and genomic breeding value estimates was significantly higher for traits with relatively high heritability over low-heritable traits (from +0.91 to +0.94 vs. +0.89). This indicates a greater contribution of genomic data to estimates for low-heritable traits with a limited number of phenotypic observations. In our opinion, the higher correlation between preliminary and final GEBV for all traits in study (from +0.60 to +0.90) compared to PA final EBV correlation (from +0.39 to +0.82) suggests the use of genomic data for predicting the breeding value of individuals. This assumption is also supported by the higher reliability of the genomic prediction (R^2^ varied from 0.36 to 0.81) versus the pedigree prediction (from 0.15 to 0.67).

If a selection trait has low heritability, this significantly hinders genetic progress, both through the conventional selection and genomic selection [41]. To achieve high accuracy for such traits, a larger number of animals with genotypes and phenotypes is required [3,4]. This was confirmed in our study for the reproduction traits. The inclusion of genomic data in the calculation of breeding value for TNB and NBA increases the reliability of the pedigree prediction for sows by 10.50 and 10.90%, respectively. For boars, the use of information about SNP markers increases the prediction’s reliability for a limited number of descendants (less than 50) almost twice (from 0.26 to 0.45 for TNB and from 0.25 to 0.44 for NBA). With an increase in the number of farrowed daughters, genomic data’s contribution to the accuracy of estimates decreases.

However, for indicators of meat and fatness traits, the superiority of genomic estimates as a selection criterion compared with parental averages is obvious, which is consistent with W. M. Muir (2007) research [4].

According to VanRaden (2008), calculations performed on simulated data showed that young animals’ reliability could be more than 60% versus 32% of the parent average value [6]. In the study of Knol E. F. et al. (2016), an increase in the accuracy of estimates was noted by 50% for piglet mortality before weaning (with very low heritability, h^2^ = 0.05) and the number of teats (with high heritability, h^2^ = 0.40) due to the use of genomic data [42]. In our study, the increase in the reliability of genomic estimates (Rel, %) in comparison with the pedigree-based estimates was 38% for the reproduction traits (from 0.26 to 0.36 for the number of all born piglets and from 0.25 to 0.35 for the number of live-born piglets) and 41–44% for meat, growth and fatness qualities (from 0.40 to 0.58 for BF1, from 0.36 to 0.50 for MD, and from 0.39 to 0.56 for Age to 100). Such a significant increase in the accuracy of genomic estimates of reproduction reveals the advantage of using genomic data for boars’ evaluation in the presence of a delimited (less than 50 heads) number of descendants. For meat and fatness traits, an increase in the prediction accuracy (pedigree with genotype) was revealed in comparison with the assessment for parents, comparable to the receipt of 4, 13 and 7 descendants included in the assessment. As for the reproduction traits, this indicator was about 20 additional descendants in the evaluation of boars and about 10 descendants in the evaluation of sows. This confirms the relatively higher weight of genomic data in the evaluation of low-inherited traits. So, in the study of Knol et al. (2016), the accuracy gain on a low-inheritance binary trait was estimated at 100 additional descendants.

## 5. Conclusions

Preliminary genomic estimates (based on pedigree and genomic data) are more relevant predictions of final genomic breeding value estimates for pigs by reproduction, meat, fatness and growth traits compared to pedigree-based predictions, which is confirmed by higher accuracy (correlation) and reliability (determination). Genomic estimates are superior to pedigree-based estimates in terms of reliability and therefore are better criteria for the selection of replacement young animals. Genomic data usage in the animal evaluation on reproduction traits is comparable to the presence of 10 to 20 additional descendants in the evaluation dataset.

## Figures and Tables

**Table 1:** The descriptive statistics of the whole dataset.

Trait	Mean	SD	Range	*N*	Number of Farms (herds)	Number of FYCG-Classes	Average Number of Record in FYCG-Class
TNB	13.91	3.77	1 to 26	9433	5	333	28.3
NBA	13.20	3.61	0 to 23				
BF1	16.23	3.57	6 to 29	41,941		467	89.8
MD	59.89	6.46	41 to 81				
Age	154.86	9.17	109 to 193				

SD—standard deviation; *N*—number of records, FYCG—class «farm-year-contemporary group»; TNB—number of all piglets born at the first farrowing, NBA—number of piglets born alive at the first farrowing, BF1—backfat thickness over 6–7 ribs, MD—muscle depth, Age—days to 100 kg.

**Table 2:** The structure of the pedigrees of the analyzed groups of individuals.

Indicator	Study Population	Reference Group
Total number of individuals	43,085	1518
Number of inbred individuals	531	0
Number of boar sires	186	83
Number of boar offspring	41,941	924
Number of dams, heads	5640	518
Number of dam offspring	41,941	924
Number of individuals with offspring	5826	601
Number of individuals without offspring	37,259	917
Number of full sibling groups	8951	203
Number of generations	6	3
Average inbreeding coefficient	0.00022	0.00000

**Table 3:** Components of variance without and with genomic information.

Trait	Values		
	Additive Variance	Residual Variance	Heritability
BLUP AM			
TNB	1.83	11.49	0.14
NBA	1.51	10.75	0.12
BF1	3.18	4.93	0.39
MD	3.26	15.37	0.17
Age	16.64	34.61	0.32
ssGBLUP			
TNB	1.69	11.61	0.13
NBA	1.40	10.82	0.11
BF1	3.34	4.87	0.41
MD	3.47	15.26	0.18
Age	17.07	34.62	0.33

TNB—number of all piglets born at the first farrowing, NBA—number of piglets born alive at the first farrowing, BF1—backfat thickness over 6–7 ribs, MD—muscle depth, and age—days to 100 kg.

**Table 4:** Correlation coefficients of final genomic and genetic estimates (EBV_WD_, GEBV_WD_) with preliminary predictions (PA_PD_, GEBV_PD_) of the validated animals.

Trait	EBV_WD_		GEBV_WD_	
	PA_PD_	GEBV_PD_	PA_PD_	GEBV_PD_
TNB	+0.80	+0.75	+0.74	+0.89
NBA	+0.82	+0.77	+0.76	+0.90
BF1	+0.56	+0.55	+0.51	+0.76
MD	+0.61	+0.54	+0.56	+0.77
Age	+0.39	+0.51	+0.38	+0.60

TNB—number of all piglets born at the first farrowing, NBA—number of piglets born alive at the first farrowing, BF1—backfat thickness over 6–7 ribs, MD—muscle depth, age—days to 100 kg; EBV_WD_, GEBV_WD_—final genetic and genomic estimates (based on whole dataset), PA_PD_, and GEBV_PD_—pedigree-based only and pedigree plus genomic data predictions (based on partial dataset).

**Table 5:** Regression coefficients (b) of final estimates (EBV_WD,_ GEBV_WD_) on predictions (PA_PD,_ GEBV_PD_) of validated animals.

Trait	EBV_WD_		GEBV_WD_	
	PA_PD_	GEBV_PD_	PA_PD_	GEBV_PD_
TNB	+1.06 (R^2^ = 0.64)	+0.71 (R^2^ = 0.57)	+1.20 (R^2^ = 0.55)	+0.99 (R^2^ = 0.79)
NBA	+1.03 (R^2^ = 0.67)	+0.59 (R^2^ = 0.48)	+1.22 (R^2^ = 0.57)	+0.98 (R^2^ = 0.81)
BF1	+0.83 (R^2^ = 0.32)	+0.60 (R^2^ = 0.30)	+0.76 (R^2^ = 0.26)	+0.88 (R^2^ = 0.58)
MD	+0.93 (R^2^ = 0.37)	+0.65 (R^2^ = 0.30)	+0.86 (R^2^ = 0.31)	+0.93 (R^2^ = 0.59)
Age	+0.54 (R^2^ = 0.15)	+0.50 (R^2^ = 0.17)	+0.53 (R^2^ = 0.15)	+0.73 (R^2^ = 0.36)

TNB—number of all piglets born at the first farrowing, NBA—number of piglets born alive at the first farrowing, BF1—backfat thickness over 6–7 ribs, MD—muscle depth, age—days to 100 kg; EBV_WD_, GEBV_WD_—final genetic and genomic estimates (based on whole dataset), PA_PD_, and GEBV_PD_—pedigree-based only and pedigree plus genomic data predictions (based on partial dataset).

**Table 6:** Reliability of predicted EBV/GEBV depending on the type of data included in the calculation *.

Reliability of Estimates Based on Data	Traits				
	BF1	MD	Age	TNB	NBA
Sires					
PA (parent average EBV)	0.43	0.38	0.42	-	-
GEBV (pedigree + genomic data)	0.56	0.49	0.55	-	-
EBV 1 (pedigree + own phenotype)	0.57	0.44	0.54	-	-
EBV 2 (EBV 1 + offspring records), *n* < N*	0.72	0.59	0.69	-	-
EBV 3 (EBV 1 + offspring records) *n* > N*	0.93	0.87	0.92	-	-
EBV4 (less than 50 offspring records)	-	-	-	0.26	0.25
EBV5 (less than 50 offspring records + genomic data)	-	-	-	0.45	0.44
EBV6 (more than 50 offspring records)	-	-	-	0.77	0.75
EBV7 (more than 50 offspring records + genomic data)	-	-	-	0.78	0.76
Dams					
PA (parent average EBV)	0.40	0.35	0.39	0.26	0.25
GEBV (pedigree + genomic data)	0.59	0.51	0.58	0.37	0.36
EBV 1 (pedigree + own phenotype)	0.57	0.44	0.53	0.35	0.33
EBV 2 (EBV 1 + offspring records), *n* < *N* *	0.64	0.50	0.60	0.38	0.37
EBV 3 (EBV 1 + offspring records) *n* > *N* *	0.76	0.61	0.73	0.50	0.48

Note: *—*n* is the actual number of offspring, N is the threshold value for the number of offspring, for boars *N* = 50, for sows *N* = 10; TNB—number of all piglets born at the first farrowing, NBA—number of piglets born alive at the first farrowing, BF1—backfat thickness over 6–7 ribs, MD—muscle depth, and age—days to 100 kg.

**Table 7:** Average breeding value (EBV) of groups formed based on PA and GEBV (by groups of validating animals).

Trait	Average EBV of Groups Formed on the Basis of *	
	PA_PD_ (Parent Average EBV)	GEBV_PD_ (Pedigree + Genomic Data)
BF1	−0.23	−0.39
MD	+0.34	+0.45
Age	−1.28	−1.59
TNB	+0.57	+0.53
NBA	+0.57	+0.51

* TNB—number of all piglets born at the first farrowing, NBA—number of piglets born alive at the first farrowing, BF1—backfat thickness over 6–7 ribs, MD—muscle depth, age—days to 100 kg; for the BF1 and age traits, negative ratings are preferred, which characterize lower values.

## Data Availability

The dataset (phenotype and genotype information) of the Large White pigs used and analysed during the current study are owned by LLC “TOPGEN” and were provided via a signed data access agreement which does not allow for data sharing.
